# Cement hardening reshapes soil microbial diversity, network stability and ecological functions of industrial area

**DOI:** 10.3389/fmicb.2026.1750259

**Published:** 2026-02-12

**Authors:** Xiaodong Hao, Hui Li, Xiaomeng Wang, Ping Zhu, Aide Sun, Qishi Luo, Xu Zhang, Zhiqun Chen, Xueduan Liu

**Affiliations:** 1 College of Resources and Environment, Linyi University, Linyi, China; 2 College of Life Science, Linyi University, Linyi, China; 3 School of Minerals Processing and Bioengineering, Central South University, Changsha, China

**Keywords:** cement hardening, community assembly, co-occurrence network, ecological function, microbial community

## Abstract

Rapid expansion of industrialization increased the area of impermeable cement layers, disrupting substance and energy exchanges between soil and atmosphere. However, the impacts of cement hardening on soil microbial community and microbiological process remain unclear. This study compared bacterial and fungal communities between cement-hardened and bare soils across five abandoned factories. The results indicated that soil cement hardening reduced exogenous nutrients input and heavy metal accumulation. Fungal alpha-diversity responded more strongly than bacterial diversity to cement hardening. Both bacterial and fungal community compositional dissimilarities shifted significantly (*p* < 0.05), driven mainly by species replacement processes (74.9 and 71.1%). Microbial network’s size and complexity increased under cement hardening, but the fungal network stability presented by robustness was decreased. Cement hardening narrowed the niche breadth of bacterial community (3.4) compared to bare soils (4.0), accompanied by an increased proportion of specialist species (increased by 52.7%). Deterministic process became more important in shaping the microbial community assembly in hardened soils, and heterogeneous selection dominated the phylogenetic variation, particularly for fungi (96%). Functionally, cement hardening enriched bacterial taxa involved in aerobic and anaerobic respiration, but reduced bacterial xenobiotics catabolism potential and inferred fungal saprophytic functions. Path modeling showed cement hardening directly altered bacterial composition and diversity, which indirectly influenced its function. In contrast, cement hardening directly influenced fungal community composition and functional diversity while indirectly modulating community diversity and functional composition. These findings offer new sights into the effect of cement hardening on soil microbial ecosystem and facilitates the ecological management of industrial areas.

## 1 Introduction

Rapid urbanization and industrialization have led to the extensive covering of natural and agricultural soils by buildings, roads, and other impervious surfaces. According to a global annual urban land cover fraction (GAULCF) dataset (2001 ~ 2020), the impervious surface area nearly doubled, with building areas increasing from 124,589 km^2^ to 206,603 km^2^ (Wang et al., 2025). As a prevalent form of land sealing, cement hardening significantly alters local climates, disrupts hydrological cycles, exacerbates environmental pollution, and diminishes soil carbon sinks (Takashi and Vu, 2000; Scalenghe and Marsan, 2009; Ding et al., 2025; Yang et al., 2025). These impervious surfaces collectively contribute to landscape fragmentation, soil degradation, and loss of microbial habitats through altered dispersal and environment conditions at the patch scale (Zhu et al., 2011; Li and Zhou, 2019). Soil microorganisms underpin key ecological processes such as biogeochemical cycles, pollutant degradation, and ecosystem stability, which makes it essential to understand the cement hardening effect on soil microbial communities for urban ecosystem management and sponge city construction.

Cement hardening impedes the exchange of gasses, water, and nutrients between soil and atmosphere, profoundly influencing microbial communities. For instance, cement hardening under pavements and roads alters total carbon, total nitrogen, carbon/nitrogen ratio, and water content, leading to reduced microbial biomass, activity, and carbon storage potential (Pereira et al., 2021). Impervious surface-induced environmental shifts such as soil pH and NH_4_^+^-N strongly reshape bacterial composition and diversity in wetlands (Yi et al., 2022). Similarly, masonry revetments affect soil organic carbon and texture, thereby inhibiting microbial denitrification in urban tidal riparian zones (Yan et al., 2019). The impact extends to symbiotic fungi, impermeable pavements alter the diversity and activity of mycorrhizal fungi and reduce mycorrhizal root colonization in urban trees (Arianna et al., 2023). These studies have examined microbial responses along broad urbanization gradients such as those based on human population density (Wang et al., 2017) or land-use transitions (Yan et al., 2016) across terrestrial and aquatic ecosystems. However, such gradient-based approaches often encompass confounding factors like varying soil types, management histories, and pollutant sources, making it difficult to isolate the specific effect of physical sealing itself. Moreover, industrial areas—characterized by intense anthropogenic pressure and extreme stressors such as soil compaction, pollutant accumulation, and nutrient imbalances—remain a critically underexplored yet significant microenvironment within urban landscapes. The unique combination of physical sealing (cement hardening) with industrial-specific stressors may induce more complex and pronounced microbial shifts than those observed in general urban settings. Consequently, a systematic and controlled assessment of how cement hardening directly influences soil microbial communities in industrial settings is still lacking.

Beyond biodiversity loss (Aronson et al., 2014; Sol et al., 2017; Xie et al., 2018), research on cement hardening should also address its effects on microbial co-occurrence patterns and assembly process. High impervious surface coverage disproportionately affects microbial connectivity by modifying soil properties such as pH, temperature, moisture, carbon/nitrogen, and nitrate levels (Zhang et al., 2020). In highly urbanized environments, co-occurrence networks are often dominated by generalist species, leading to structurally simplified and unstable communities (Landi et al., 2018). Intensive land use similarly reduces bacterial network complexity and shifts hub taxa from copiotrophs to oligotrophs (Karimi et al., 2019). These topological changes can reshape microbial community structure, assembly mechanisms, and ecosystem functioning. Nevertheless, the specific role of cement hardening in modulating microbial co-occurrence patterns, assembly processes, and ecological outcomes in industrial environments remains unclear.

In this study, we used high-throughput sequencing of 16S rRNA and ITS genes to compare soil bacterial and fungal communities between cement-hardened and bare soils across five industrial sites with varying production types. A paired sampling design was employed to isolate the effect of cement hardening from pre-existing land-use history. We aimed to address the following questions: (i) the responses of bacterial and fungal diversity to cement hardening; (ii) the restructuring of microbial co-occurrence networks and community assembly processes caused by cement hardening; and (iii) the key drivers reshaping the structure and function of bacterial and fungal communities under cement-sealed conditions.

## 2 Materials and methods

### 2.1 Study sites and soil sampling

**Figure 1:**
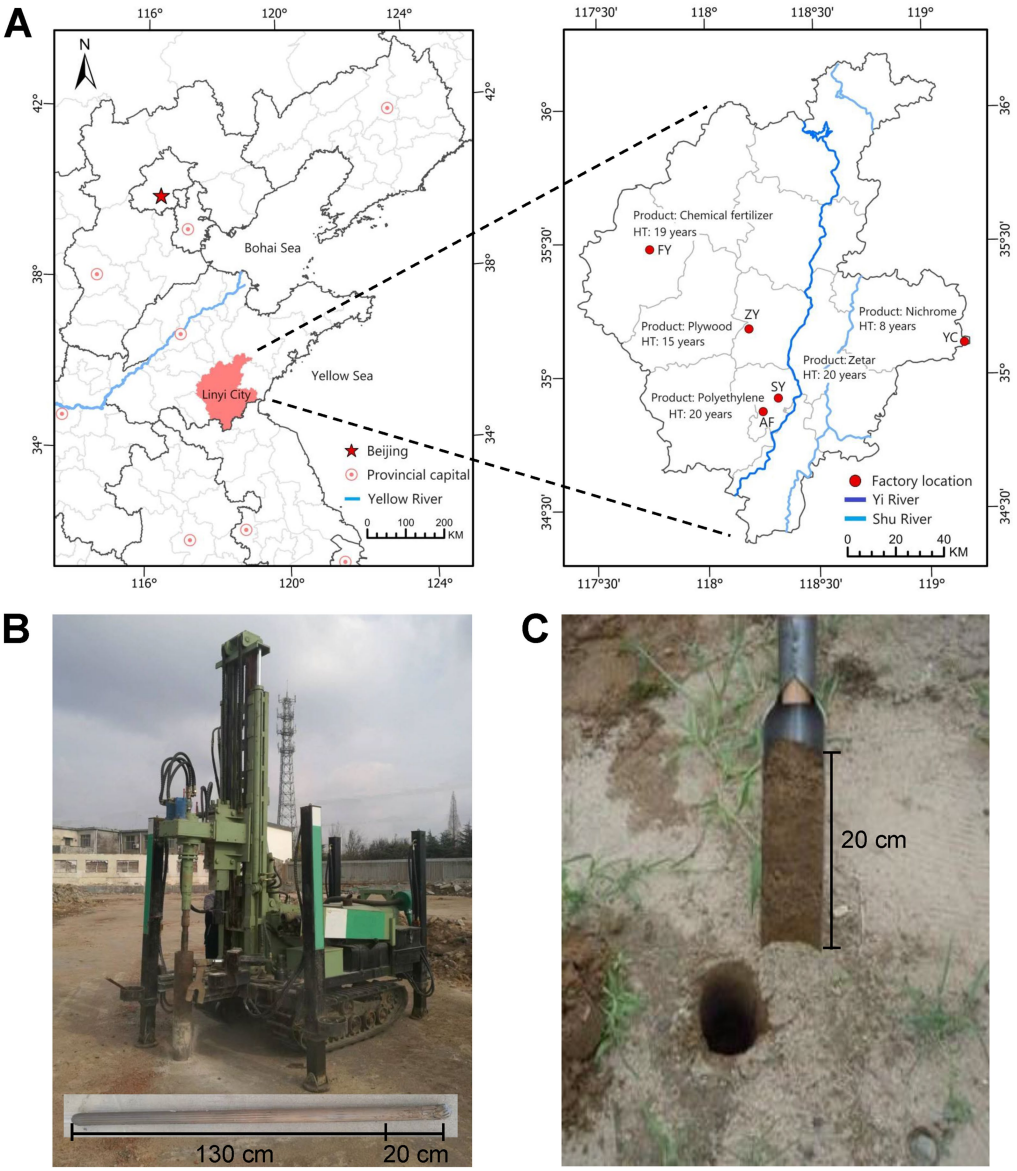
Soil sampling sites and methods. **(A)** Locations of factory sites in Linyi City. **(B)** Sample collection of cement-hardened soils (CH). **(C)** Sample collection of bare soils (CK). HT, Cement-hardened time.

The soil sampling sites were located at Linyi City (34°22′ ~ 36°13′N, 117°24′ ~ 119°11′E) of Shandong Province, Eastern China (Figure 1A). As a national manufacturing base in China, Linyi City has a variety of industrial production categories. This area experiences a temperate monsoon climate with a mean annual precipitation of 799.9 mm (~2.2% occurs during October to December) and a mean annual temperature of 15.0 °C.

To capture the natural heterogeneity of industrial environments and robustly test the generality of the cement-hardening effect on soil ecosystems, a stratified selection approach was employed. We selected five factory sites representing two major industrial categories (chemical manufacturing and synthetic materials & processing) in the region, each defined by distinct production activities and associated potential soil contamination profiles. This design ensured our findings were not idiosyncratic to a single factory type but reflected broader patterns across industrial landscapes. All sites were located within Linyi City to control for macro-climatic and broad soil-type variations. Critically, within each factory, the paired plot design was used to isolate the effect of cement hardening from pre-existing land-use history. The selected factories were: Fenyan Co., LTD. (FY, 35°28′N, 117°44′E), which produced chemical fertilizer in Pingyi County, representing the chemical manufacturing category; Zhuoyue Wood Industry Co., LTD. (ZY, 35°12′N, 118°23′E, plywood) and Anfen Chemical Co., LTD. (AF, 35°08′N, 118°26′E, polyethylene) in Lanshan and Luozhuang Districts, respectively, representing the synthetic materials and processing category; Yaocheng Nickel-Chromium Alloy Co., LTD. (YC, 35°06′N, 119°09′E, nichrome) in Ju′nan County, also under chemical manufacturing with a focus on metallurgy; and Shenyan Group Co., LTD. (SY, 34°54′N, 118°18′E, zetar) in Luozhuang District, representing synthetic materials & processing.

Soil sampling was conducted in November 2023 across five factories in Linyi City using a paired sampling design to isolate the effect of cement hardening. Within each factory, six pairs of adjacent sampling plots (5 m × 5 m each) were established, uniformly distributed across the factory grounds. Each pair consisted of one cement-hardened (CH) plot and one adjacent unsealed bare soil (CK) plot (within 1 ~ 3 m), both sharing identical pre-sealing land-use history. For CH plots, the cement surface debris was first removed by brushing and rinsing with distilled water to minimize surface contamination of subsurface sample. A crawler-mounted core drill (SE3000, Qianhe Environment, China) cooled with sterile distilled water was then used to cut through the cement layer and extract a 20-cm diameter cement core. After manual removal of the cement core, the underlying undisturbed soil surface was exposed. A sterilized polyethylene tube (6.0 cm diameter) was inserted vertically into the soil profile to collect a subsurface soil core from 0 ~ 20 cm depth. For adjacent CK plots, soil cores (0 ~ 20 cm depth, 6.0 cm diameter) were collected vertically using a manual T-type soil sampler. The sampler was sterilized with 75% ethanol between plots. In each plot, three soil cores were collected from random locations and homogenized to form one composite sample per plot. A total of 60 soil samples were obtained, comprising 30 CK samples and 30 CH samples. All soil samples were passed through a 2 mm mesh to remove stones and plant residues, and were brought back to the laboratory. A portion of the soil samples was air-dried for soil physical–chemical analysis, and the second portion was stored at −80 °C for soil microbial DNA extraction.

### 2.2 Soil physical–chemical analysis

Soil moisture was determined gravimetrically by comparing the weights of fresh soils before and after ovendrying at 105 °C until constant weight. Soil pH and electrical conductivity (EC) values were measured using a soil-to-water ratio of 1:2.5 and 1:5 (w/v) suspension by a pH meter (BPH-220, Bell Instrument, China) and a conductometer (DDSJ-308F, Leici Instrument, China). Soil organic matter (OM) was estimated using the loss on ignition method. Samples were dried at 105 °C for 24 h prior to weighing, heated at 550 °C for 6 h and reweighed to determine the loss of OM by weight as a percentage. Total carbon and nitrogen contents were determined by the Vario EL III elemental analyzer (El Vario analyzer, Elementar, Germany). Soil total phosphorus, total potassium, and total heavy metals (chromium, nickel, plumbum, and arsenic) contents were determined using the inductively coupled plasma-optical emission spectrometer (ICP-OES) technique (Optima 5300DV, PerkinElmer, USA) after digesting by an acid mixture of nitric acid (HNO_3_), hydrofluoric acid (HF), and perchloric acid (HClO_4_) (10:5:2, v/v) using the electric heating plate (XJS20-42, Laboratory Instrument, China). It was noted that the pretreatment procedures differed among analyzes according to the specific property measured: state variables such as moisture, pH, and EC were analyzed on fresh soils; total elemental contents required acid digestion; and organic matter was determined via combustion. All samples for any given analysis were subjected to the identical pretreatment and analytical protocol to guarantee comparability across the CH and CK groups.

### 2.3 Amplicon sequencing

Total genome DNA was extracted from 500 mg of frozen soil samples using CTAB-SDS method (Zhou et al., 1996). DNA quality and purity was checked with 260/280 and 260/230 nm wavelength ratios using the NanoDrop™ (Thermo Scientific, Massachusetts, USA). The primer sets of 341F (5’-CCTAYGGGRBGCASCAG-3’) and 806R (5’-GGACTACNNGGGTATCTAAT-3’) were used to amplify the V3 ~ V4 region of bacterial 16S rRNA gene (Berg et al., 2012). The primer pairs of ITS1-1F-F (5’-CTTGGTCATTTAGAGGAAGTAA-3’) and ITS1-1F-R (5’-GCTGCGTTCTTCATCGATGC-3’) were selected to amplify the ITS1-1F region of fungal rRNA gene (Li et al., 2021). The 30 μL polymerase chain reaction (PCR) mix consisted of 13 μL sterilized H_2_O, 15 μL High-Fidelity PCR Master Mix (New England Biolabs, USA), 0.5 μL each primer, and 1 μL template DNA (10 ng). DNA samples were amplified using the following thermal cycling conditions in three replicate runs: initial denaturation at 98.°C for 1 min, followed by 30 cycles of denaturation at 98.°C for 10 s, annealing at 50.°C for 30 s, and elongation at 72.°C for 60 s, and a final extension step at 72.°C for 5 min. Duplicate PCR products mixed with the same volume of 1 × loading buffer (contained SYB green) were pooled and quality checked on 2% agarose gel. The amplicons with bright main strip between 400 ~ 600 bp were chosen and purified using the E. Z. N. A.™ Gel Extraction Kit (Omega Bio-Tek Inc., USA). Sequencing libraries were generated and library quality was assessed on the Qubit@ 2.0 Fluorometer (Thermo Scientific, USA) and Agilent Bioanalyzer 2,100 system (Agilent Technologies, Palo Alto, USA). At last, the library was sequenced on an Ion S5™ XL platform (E-gene, Shenzhen, China) and 400 bp/600 bp single-end reads were generated.

Microbiome bioinformatics were performed with quantitative insights into microbial ecology 2 (QIIME 2) with slight modification according to the official tutorials. Briefly, raw sequence data were demultiplexed using the demux plugin following by primers cutting with cutadapt plugin (Martin, 2011). Sequences were then quality filtered, denoised, merged and chimera removed using the divisive amplicon denoising algorithm 2 (DADA 2) plugin (Callahan et al., 2016). Non-singleton amplicon sequence variants (ASVs) were aligned with mafft (Katoh et al., 2002) and used to construct a phylogeny with fasttree2 (Price et al., 2010). Taxonomy was assigned to ASVs using the classify-sklearn naïve Bayes taxonomy classifier in feature-classifier plugin (Bokulich et al., 2018) against the Greengenes (McDonald et al., 2012) and UNITE (Abarenkov et al., 2010) databases for bacterial and fungal sequences.

### 2.4 Microbial ecological network analysis

Microbial co-occurrence networks were constructed by the Molecular Ecological Network Analyzes pipeline (MENAP, http://ieg2.ou.edu/MENA/) based on the Spearman’s correlation matrices (Wen et al., 2022). The correlation thresholds of *r* threshold > 0.6 and *p* threshold < 0.05 were used to build the four networks of soil bacterial and fungal communities from CK and CH samples. Network topological properties, including connectance, average degree, average path length, diameter, mean clustering coefficient, Centralization, and modularity were calculated (Deng et al., 2012). The robustness was performed to evaluate the community stability of CK and CH network. In this study, a certain proportion of nodes was randomly removed to simulate random species removal, and certain numbers of module hubs were removed to simulate targeted removal. The robustness of the network was measured by the proportion of nodes remaining after deletion of the specified species.

### 2.5 Microbial community assembly mechanisms

Niche breadth was used to measure habitat specialization patterns among ASVs using the Spaa R package. The higher niche breadth values of ASVs represented taxa that were widely distributed on a large scale with relatively even abundance, while those with lower values indicated taxa occurred in fewer habitats with uneven distribution patterns. The niche breadth values of CK and CH soil samples were calculated based on species abundance and evenness.

ASVs occurrences were simulated to determine the niche specialization through 1,000 permutations using quasiswap permutation algorithms implemented in the EcolUtils R package. Generalist species typically exhibited broader fundamental niches compared to specialists. In this study, ASVs were classified as generalists or specialist if their observed occurrence exceeded the upper or fell below the lower 95% confidence interval. ASVs with niche breadth values within the 95% confidence interval range were categorized as neutral taxa.

To assess the importance of deterministic mechanisms from stochastic mechanisms in microbial community assembly, the *β* nearest taxon index (*β*NTI) was calculated based on taxonomic metrics using the NST R package. Homogeneous selection (*β*NTI < −2) and heterogeneous selection (*β*NTI > + 2) were used to describe the deterministic assembly process of CK and CH soil microorganisms.

### 2.6 Microbial functional prediction analysis

Potential bacterial functions among soil microbiota in CK and CH samples were predicted by BugBase (Ward et al., 2017), phylogenetic investigation of communities by reconstruction of unobserved states 2 (PICRUSt2) (Douglas et al., 2020), and FAPROTAX (Louca et al., 2016) softwares, and fungal functions were predicted based on fungal taxa using the FUNGuild database (Nguyen et al., 2016). BugBase provided an algorithm that predicted organism-level coverage of functional pathways as well as biologically interpretable phenotypes. PICRUSt2 contained an updated and larger database of gene families and reference genomes, provided interoperability with any denoising algorithm. PICRUSt2 inferred metagenomic potential based on phylogenetic placement and reference genomes, which might be affected by horizontal gene transfer and database gaps. FAPROTAX was a database that mapped prokaryotic clades (e.g., genera or species) to established metabolic or other ecologically relevant functions, using the current literature on cultured strains. FUNGuild was an open annotation tool for parsing fungal community datasets by ecological guild. FUNGuild assigned ecological roles based on published literature, but fungal nutritional modes were often facultative, and assignments were taxonomy-dependent. Firstly, the rarified taxon tables were first translated into function tables, based on taxon-function annotations in the BugBase, PICRUSt, FAPROTAX, and FUNGuild database. Then the relative abundance of each functional group was calculated based on the number of sequences per sample.

### 2.7 Statistical analysis

The alpha-diversity indices of Chao1 and Shannon index were calculated to measure bacterial and fungal community structures, and richness, Pielou’s evenness, and Shannon index were used to estimate bacterial and fungal community functions. Non-metric multidimensional scaling (NMDS) ordination and principal component analysis (PCA) based on the Bray−Curtis distance metrics were used to present the heterogeneities of microbial community structures and functions between the CK and CH soil samples. The Anosim analysis was performed to show the difference significance. The contributions of species replacement and species richness difference on the total beta-diversity of microbial community were analyzed using adespatial R package (Shen et al., 2020). The significant differences in soil physicochemical factors, alpha-diversity indices of microbial community structure and function, and relative abundances of dominant microbial phyla, genera, and functional traits between the CK and CH soils were analyzed by Wilcoxon signed-rank test. The significant effects of factory location and cement-hardened time on each soil property were estimated by One-way ANOVA. Canonical correlation analysis (CCA) and Mantel tests were used to link the microbial community structures to soil variables. The soil variables significantly (*p* < 0.05) associated with microbial community changes were selected for CCA plotting. Pearson correlation analysis was used to test the interrelation coefficients of dominant bacteria and fungi at the phylum and genus levels and soil environmental variables. Partial least square path modeling (PLSPM) using packages amap, shape, diagram, and plspm was constructed to analyze the effects of cement hardening on soil variables, microbial community composition and diversity, and microbial function composition and diversity.

## 3 Results

### 3.1 Soil environmental variables

Soil physicochemical properties exhibited considerable variations across the five factory sites. Soil variables from the Yaocheng Nickel-Chromium Alloy Co., LTD. (YC, nichrome production) and Fenyan Co., LTD. (FY, chemical fertilizer production) differed visibly from those of the other three factories (Supplementary Figure S1). Notably, the YC site showed the lowest levels of soil organic matter (OM), total carbon (TC), and total nitrogen (TN), but the highest concentrations of total nickel (TNi) and total chromium (TCr), which reflected the inherent regional variations in industrial history and baseline conditions (Table S1). The soil physicochemical properties under cement hardening (CH) tended to deviate from those of the bare soils (CK) as indicated by principal component analysis (PCA). In detail, cement hardening significantly (*p* < 0.05) increased soil moisture by 45.2% and decreased soil contents of OM, TC, and total potassium (TK) by 3.8 g/kg, 10.0 g/kg, and 1.3 g/kg, respectively (Table 1). The significant (*p* < 0.05) effects of factory location and cement-hardened time on soil electrical conductivity (EC) value and soil nutrients such as OM, TC, TN, total phosphorous (TP), and TK were observed. Soil EC and pH values were higher in CH soils than that of CK soils, with an increase of 95.1 μS/cm and 0.2 units. In addition, the impact of cement hardening on soil TN was consistent with TC, but increased soil available nitrogen (AN) level by 16.6%. Although no significant sealed effects were observed on total heavy metal levels, cement hardening decreased the soil contents of TCr, TNi, total plumbum (TPb), and total arsenic (TAs).

**Table 1:** Soil physicochemical properties of bare soils (CK) and cement-hardened soils (CH) (*n* = 30).

Item	CK	CH	FL (*p*)	HT (*p*)
Moisture, %	11.5 ± 4.6	**16.7 ± 4.2**	0.311	0.154
EC, μS/cm	396.8 ± 198.3	491.9 ± 281.0	**0.027**	**0.010**
pH	7.8 ± 0.2	8.0 ± 0.4	0.575	0.895
OM, g/kg	**22.5 ± 12.8**	18.7 ± 20.8	**0.015**	**0.004**
TC, g/kg	**24.9 ± 21.7**	14.9 ± 17.6	**0.016**	**0.005**
TN, g/kg	0.9 ± 0.4	0.8 ± 0.4	**0.046**	**0.047**
AN, mg/kg	62.1 ± 18.7	72.4 ± 26.0	0.309	0.353
TP, g/kg	0.6 ± 0.2	0.6 ± 0.3	**0.002**	**<0.001**
TK, g/kg	**21.0 ± 6.3**	19.7 ± 5.5	**<0.001**	**<0.001**
TCr, mg/kg	151.2 ± 152.2	98.5 ± 46.9	0.335	0.275
TNi, mg/kg	187.4 ± 397.3	42.3 ± 13.7	0.463	0.267
TPb, mg/kg	33.7 ± 10.6	28.8 ± 3.9	0.281	0.164
TAs, mg/kg	10.2 ± 5.8	8.4 ± 2.9	0.053	0.087

EC, electrical conductivity; OM, organic matter; TC, total carbon; TN, total nitrogen; AN, available nitrogen; TP, total phosphorous; TK, total potassium; TCr, total chromium; TNi, total nickel; TPb, total plumbum; TAs, total arsenic; FL, factory location; HT, cement-hardened time. Bold fonts in CK and CH columns represent the significantly (*p* ≤ 0.05) higher means according to Wilcoxon signed-rank test. Bold fonts in FL and HT columns represent the significant effects (*p* ≤ 0.05) of factory location and cement-hardened time on each soil property according to One-way ANOVA.

### 3.2 Changes in microbial community structure

**Figure 2:**
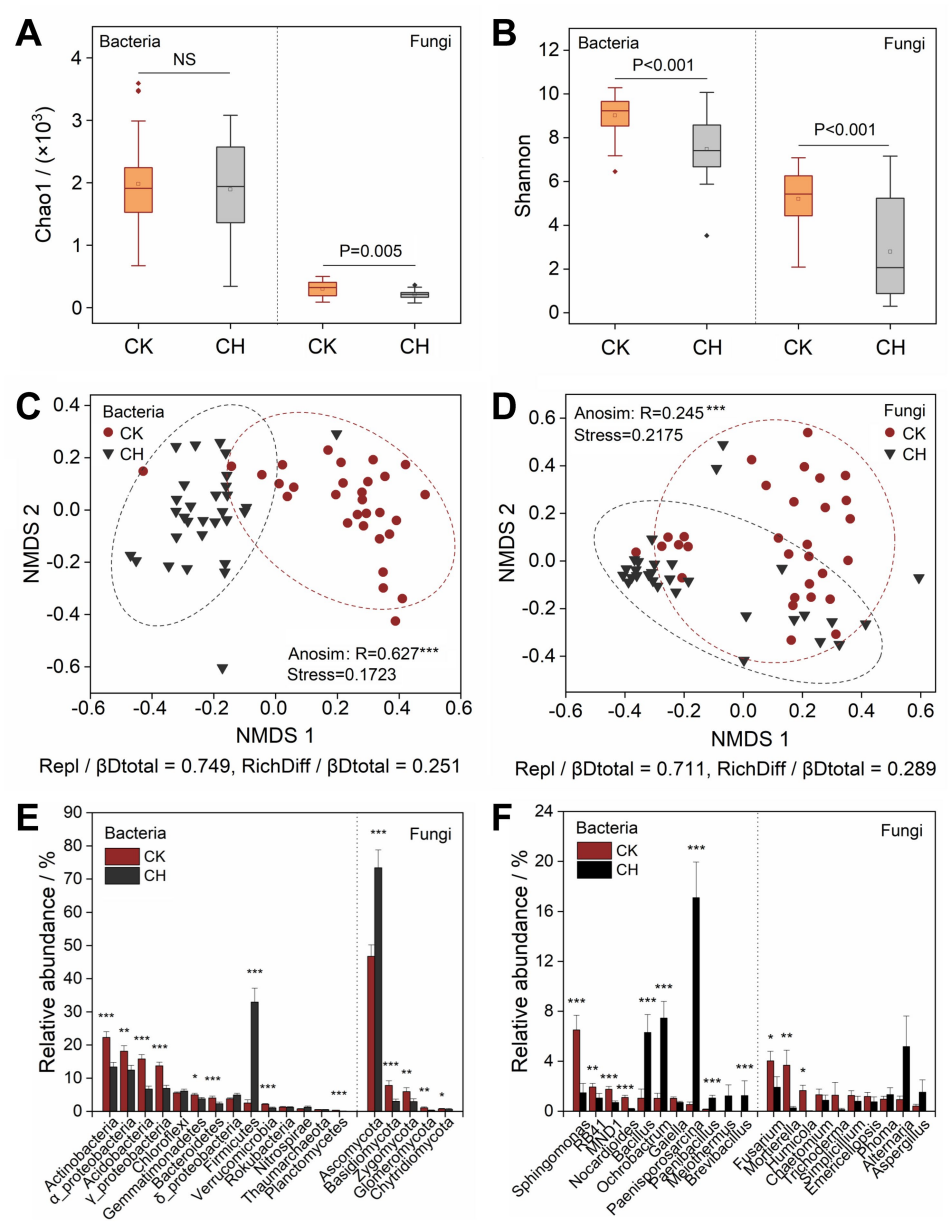
Microbial community structures of bare soils (CK) and cement-hardened soils (CH). **(A, B)** Soil bacterial and fungal community alpha-diversities indicated by Chao1 and Shannon indices. **(C, D)** NMDS analysis of bacterial and fungal community structures based on the Bray−Curtis distance metrics. **(E, F)** Relative abundances of dominant bacterial and fungal phyla and genera. Similarity test (Anosim) shows the significant (*p* ≤ 0.05) difference in soil microbial community structures of CK and CH groups. Repl, species replacement. RichDiff, species richness difference. βDtotal, total beta-diversity. *p* value (≤ 0.05) and asterisk represent the significant difference according to Wilcoxon signed-rank test. ***p ≤ 0.001, **p ≤ 0.01, and *p ≤ 0.05. NMDS, non-metric multidimensional scaling.

There was no significant (*p* > 0.05) difference in bacterial Chao1 value between CK and CH soils (Figure 2A), but the Shannon indexes decreased as the soils were sealed with cement (Figure 2B). Both the fungal richness and diversity of the CH soils were significantly (*p* < 0.005) lower than those of the CK soils (Figure 2B). Non-metric multidimensional scaling (NMDS) plots revealed the divergent bacterial and fungal communities after the soil seal (Figures 2C,D), and the Anosim tests further indicated the bacterial (*R* = 0.627, *p* < 0.001) and fungal (*R* = 0.245, *p* < 0.001) community structures of the CK soils were highly dissimilar to that of the CH soils. The beta-diversity decomposition analysis showed that the species replacement processes dominated the bacterial (74.9%) and fungal (71.1%) community compositional dissimilarities among all study samples, while the richness difference processes only contributed 25.1 and 28.9% on average. Cement hardening significantly (*p* < 0.05) decreased the relative abundances of *Actinobacteria*, *Proteobacteria*, *Acidobacteria*, *Gemmatimonadetes*, *Bacteroidetes*, *Verrucomicrobia*, and *Planctomycetes* in the bacterial community, but increased (*p* < 0.001) the relative abundance of *Firmicutes* in the CK soils (Figure 2E). For the fungal community, the decreased relative abundances under cement seal were observed for *Basidiomycota*, *Zygomycota*, *Glomeromycota*, and *Chytridiomycoa*, while increased the relative abundance of *Ascomycota*. At the genus level, cement hardening led to a significant (*p* < 0.05) decrease in the relative abundances of bacterial genera *Sphingomonas*, *RB41*, *MND1*, and *Nocardioides*, as well as fungal genera *Fusarium*, *Mortierella*, and *Humicola*. Conversely, it significantly (*p* < 0.001) increased the abundances of bacterial genera including *Bacillus*, *Ochrobactrum*, *Paenisporosarcina*, *Paenibacillus*, and *Brevibacillus*.

### 3.3 Microbial co-occurrence networks

Soil cement hardening distinctly changed the topological properties of soil microbial co-occurrence networks, especially for the fungal network (Table 2). The bacterial network of CH soils was greatly larger (e.g., more nodes, links, and diameter) than that of CK soils. Although the nodes numbers of the fungal network were slightly decreased from 382 (CK) to 375 (CH), the links number and diameter were 2.3 times and 1.2 times higher that of CK soils, respectively. In addition, more complex (e.g., higher connectance, average degree, and average clustering coefficient) CH soil networks of both bacterial and fungal communities were observed compared to CK soils. Conversely, the modularity net and modularity random of the microbial networks were decreased with the cement seal of the soil.

**Table 2:** Microbial co-occurrence network topological properties of bare soils (CK) and cement-hardened soils (CH).

Network property	Bacteria		Fungi	
	CK	CH	CK	CH
Nodes	369	378	382	375
Links	3,277	4,583	2,328	5,296
Diameter	6.03	7.22	8.53	10.24
Connectance	0.05	0.06	0.03	0.08
Average degree	17.76	24.25	12.19	28.25
Average path length	3.69	3.81	5.17	4.85
Mean clustering coefficient	0.55	0.64	0.59	0.79
Centralization degree	0.14	0.16	0.1	0.12
Centralization betweenness	0.10	0.09	0.17	0.21
Centralization closeness	0.02	0.23	0.02	0.06
Relative modularity	2.59	2.93	2.02	4.35
Modularity net	0.6	0.55	0.66	0.66
Modularity random	0.17	0.14	0.22	0.12

**Figure 3:**
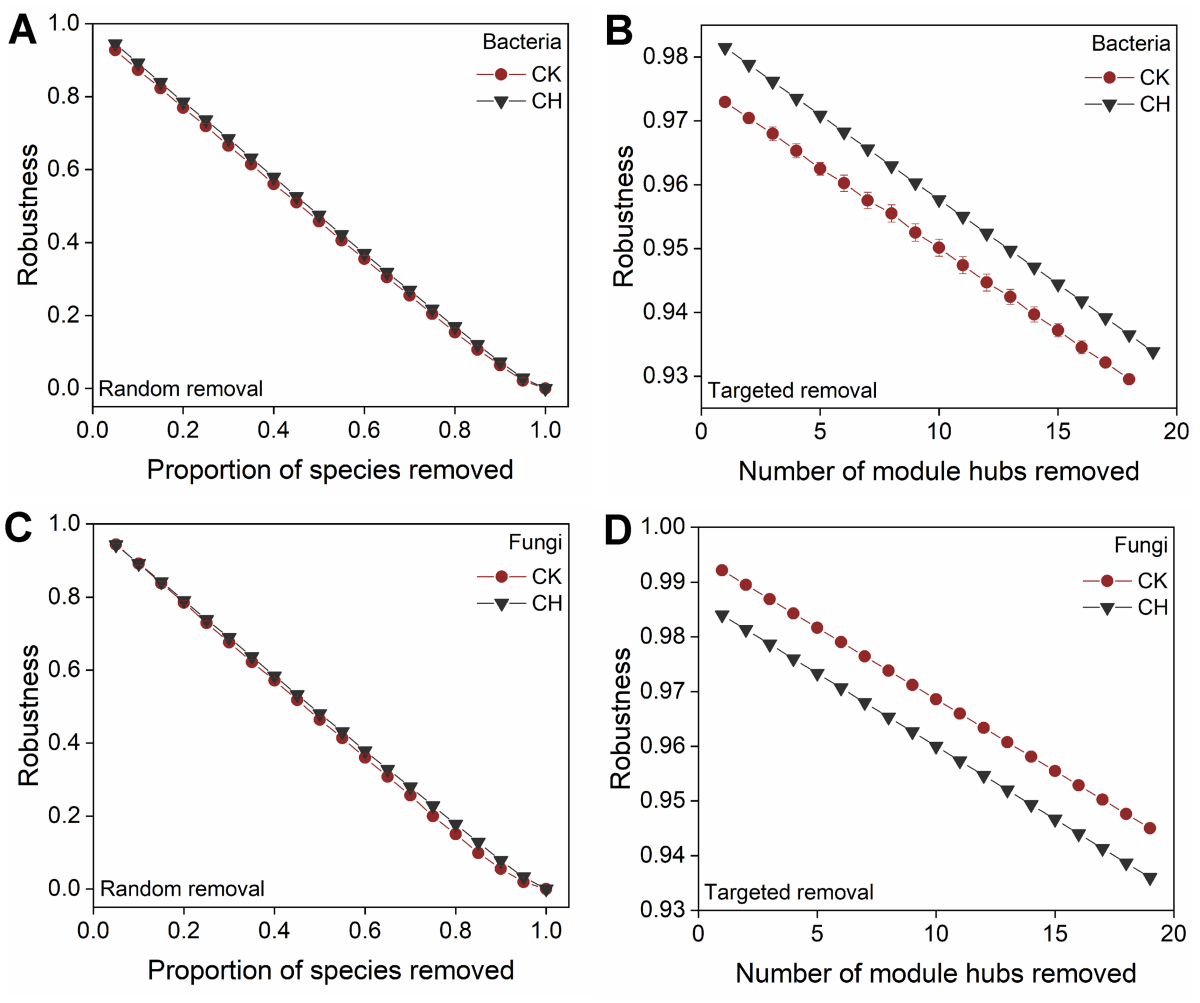
Stability of microbial networks measured by robustness. **(A, B)** Robustness represents the proportion of taxa that remained with the taxa randomly and targetedly removed from bacterial network. **(C, D)** Robustness represents the proportion of taxa that remained with the taxa randomly and targetedly removed from fungal network.

Both random and targeted taxa removal were simulated to evaluate the robustness of microbial networks, quantified as their resistance to node loss (Figure 3). Under random taxa loss, bacterial and fungal networks in cement-sealed soils exhibited robustness comparable to those in bare soils (Figures 3A,C). Notably, based on targeted taxa removal, the bacterial network demonstrated higher robustness in CH soils compared to CK soils (Figure 3B). In contrast, fungal networks showed an inverse response pattern, with CH soils demonstrating markedly reduced robustness relative to CK soils (Figure 3D).

### 3.4 Microbial community assembly mechanisms

**Figure 4:**
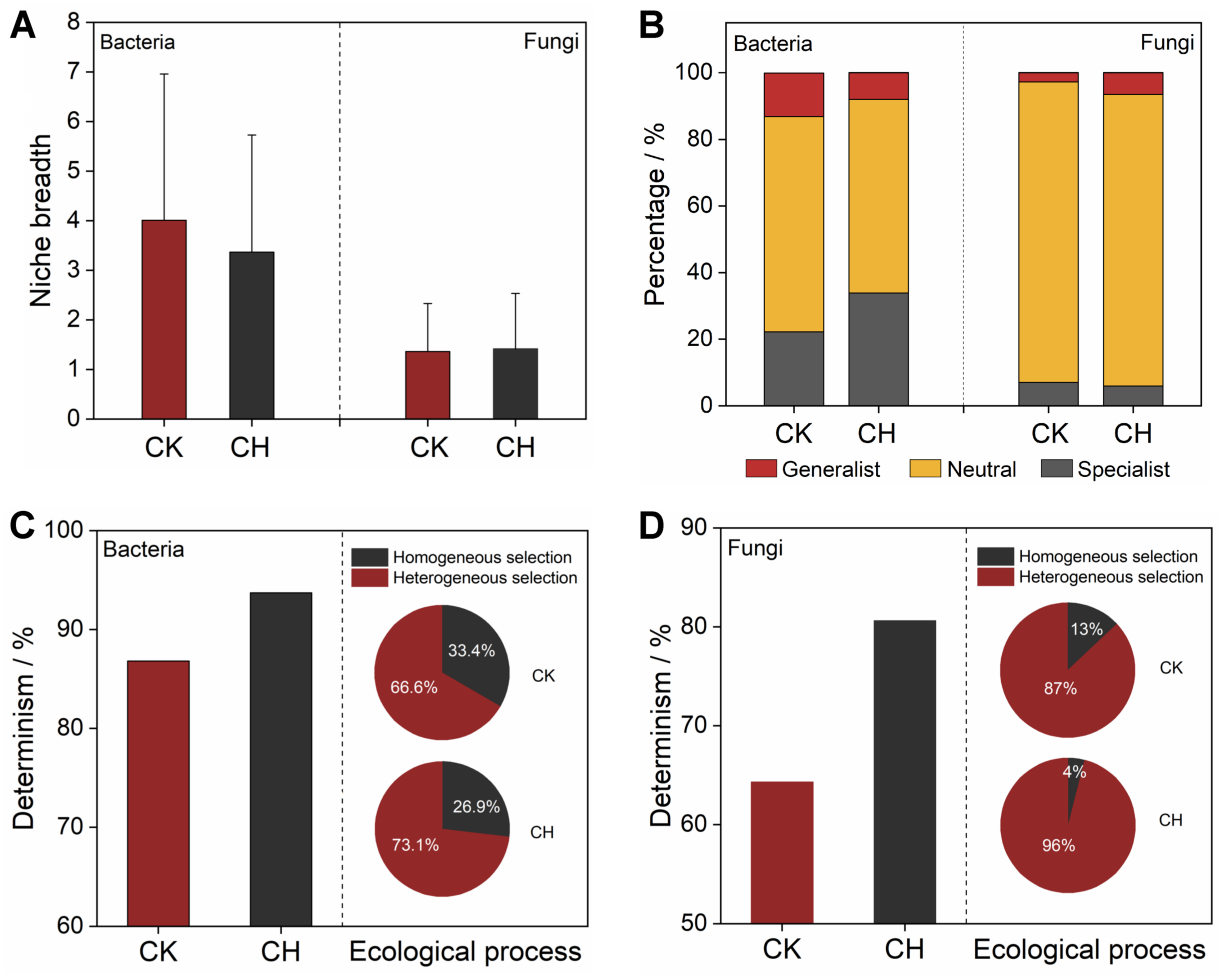
Microbial community assembly of bare soils (CK) and cement-hardened soils (CH). **(A)** Niche breadth of bacterial and fungal ASVs in CK and CH soils. **(B)** Percentages of bacteria and fungi in generalist species, neutral species, and specialist species of CK and CH soils. **(C, D)** Contributions of ecological assembly process governing bacterial and fungal communities in CK and CH soils. ASVs, amplicon sequence variants.

The niche breadth was applied to assess microbial habitat specialization patterns following cement hardening. Cement hardening distinctly reduced the niche breadth of bacterial community (Figure 4A), accompanied by a marked increase in specialist species proportion and a corresponding decrease in generalist species (Figure 4B). In contrast, the fungal community exhibited only marginal elevation in niche breadth under cement-hardened conditions. The percentage of generalist fungal species increased from 2.8% (CK) to 6.5% (CH), but neutral species remained the dominant component of the fungal community in both CK and CH soils. Deterministic processes exerted a stronger influence on the assembly of both bacterial and fungal communities in CH soils compared to CK soils (Figures 4C,D). Cement hardening significantly enhanced heterogeneous selection process in microbial community assembly. Particularly for fungi, the deterministic heterogeneous selection process accounted for nearly all (96%) of the observed phylogenetic variation in CH soils, which presented a larger extent than observed in bacteria.

### 3.5 Profiles of microbial community functional prediction

**Figure 5:**
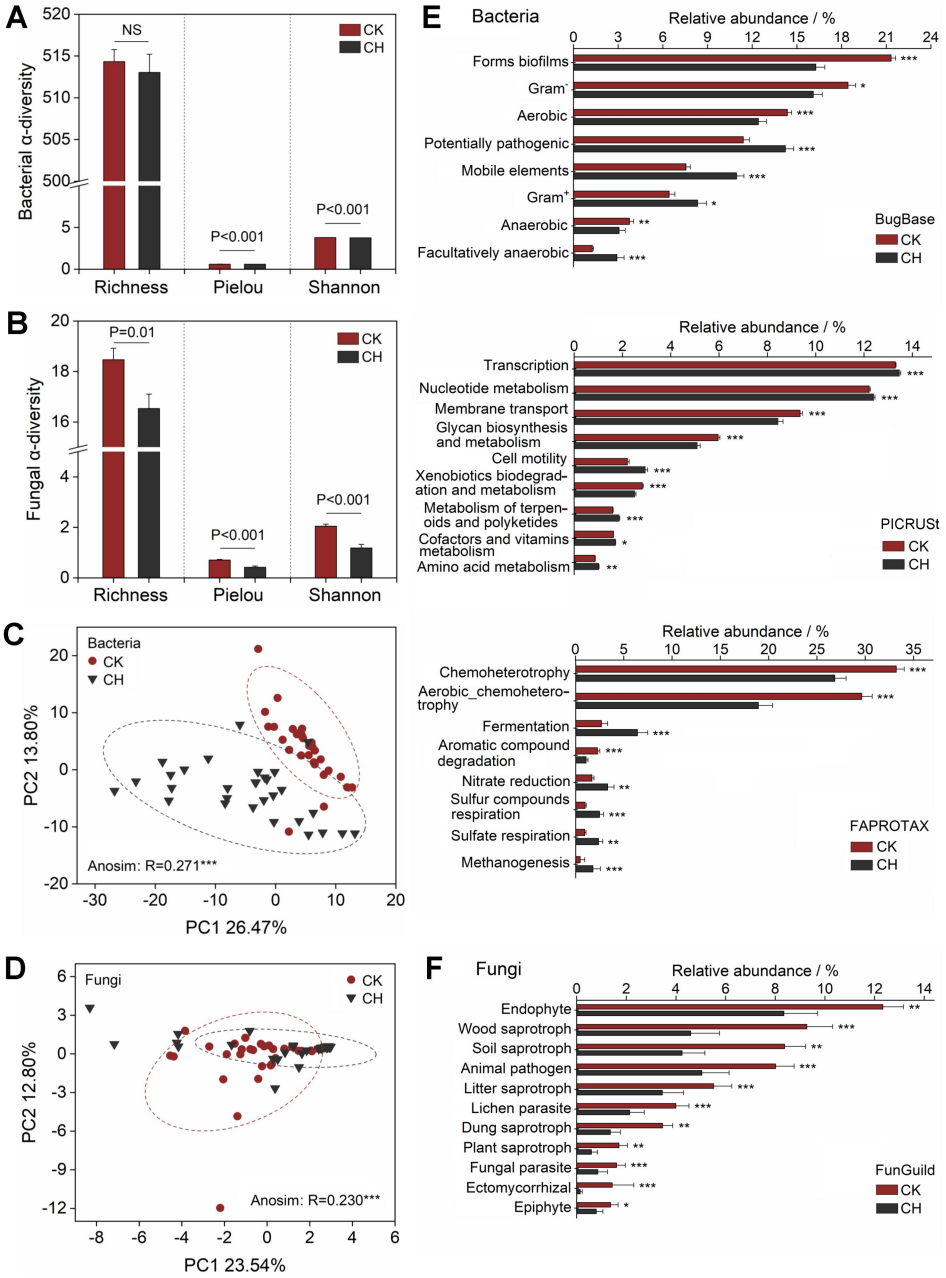
Microbial community functions of bare soils (CK) and cement-hardened soils (CH). **(A, B)** Functional alpha-diversities of bacterial and fungal community indicated by richness, Pielou, and Shannon indices. **(C, D)** PCA of bacterial and fungal community functions based on the Bray−Curtis distance metrics. **(E, F)** Relative abundance of bacterial and fungal functions based on the functional predictions. Similarity test (Anosim) shows the significant (*p* ≤ 0.05) difference in soil microbial community functions of CK and CH groups. *p* value (≤ 0.05) and asterisk represent the significant difference according to Wilcoxon signed-rank test. ***p ≤ 0.001, **p ≤ 0.01, and *p ≤ 0.05. PCA, principal component analysis.

The putative metabolic or ecological functions of bacterial and fungal amplicon sequence variants (ASVs) were comparatively analyzed between CK and CH soils. Bacterial α-diversity (richness) remained unaffected by cement hardening, while both the Pielou evenness and Shannon diversity indices exhibited significant reductions (*p* < 0.001) (Figure 5A). In contrast, fungal functional α-diversity demonstrated a pronounced decline across all metrics, including richness, Pielou evenness, and Shannon diversity, in response to cement hardening (Figure 5B). PCA revealed that significant divergences (*p* < 0.001) in the functional composition of both bacteria and fungi were observed under cement-hardened conditions (Figures 5C,D).

As predicted by the Bugbase analysis, soil cement hardening significantly decreased the relative abundances of bacterial functional groups associated with forms biofilms, Gram^−^ bacteria, and both aerobic and anaerobic taxa, while enriched potentially pathogenic bacteria, mobile elements, Gram^+^ bacteria, and facultatively anaerobes (Figure. 5E). Phylogenetic investigation of communities by reconstruction of unobserved states 2 (PICRUSt2) analysis suggested that cement hardening exerted significant negative effects (*p* < 0.001) on pathways related to membrane transport, glycan biosynthesis and metabolism, and xenobiotics biodegradation and metabolism. In contrast, functional groups associated with transcription, nucleotide metabolism, cell motility, terpenoid and polyketide metabolism, cofactor and vitamin metabolism, and amino acid metabolism were markedly enhanced. FAPROTAX annotation demonstrated a pronounced decline (*p* < 0.001) in chemoheterotrophy, aerobic chemoheterotrophy, and aromatic compound degradation in CH soils compared to CK soils. Conversely, metabolic pathways linked to fermentation, nitrate reduction, sulfur compounds/sulfate respiration, and methanogenesis exhibited an increasing trend. Fungal functions were also significantly (*p* < 0.05) impacted, with cement hardening reducing the relative abundances of dominant ecological roles such as endophyte, wood/soil/litter/plant/dung saprotrophs, animal pathogen, lichen parasite, fungal parasite, ectomycorrhizal fungi, and epiphyte across all sampling sites (Figure 5F).

### 3.6 Links between soil variables and microbial community structure and function

**Figure 6:**
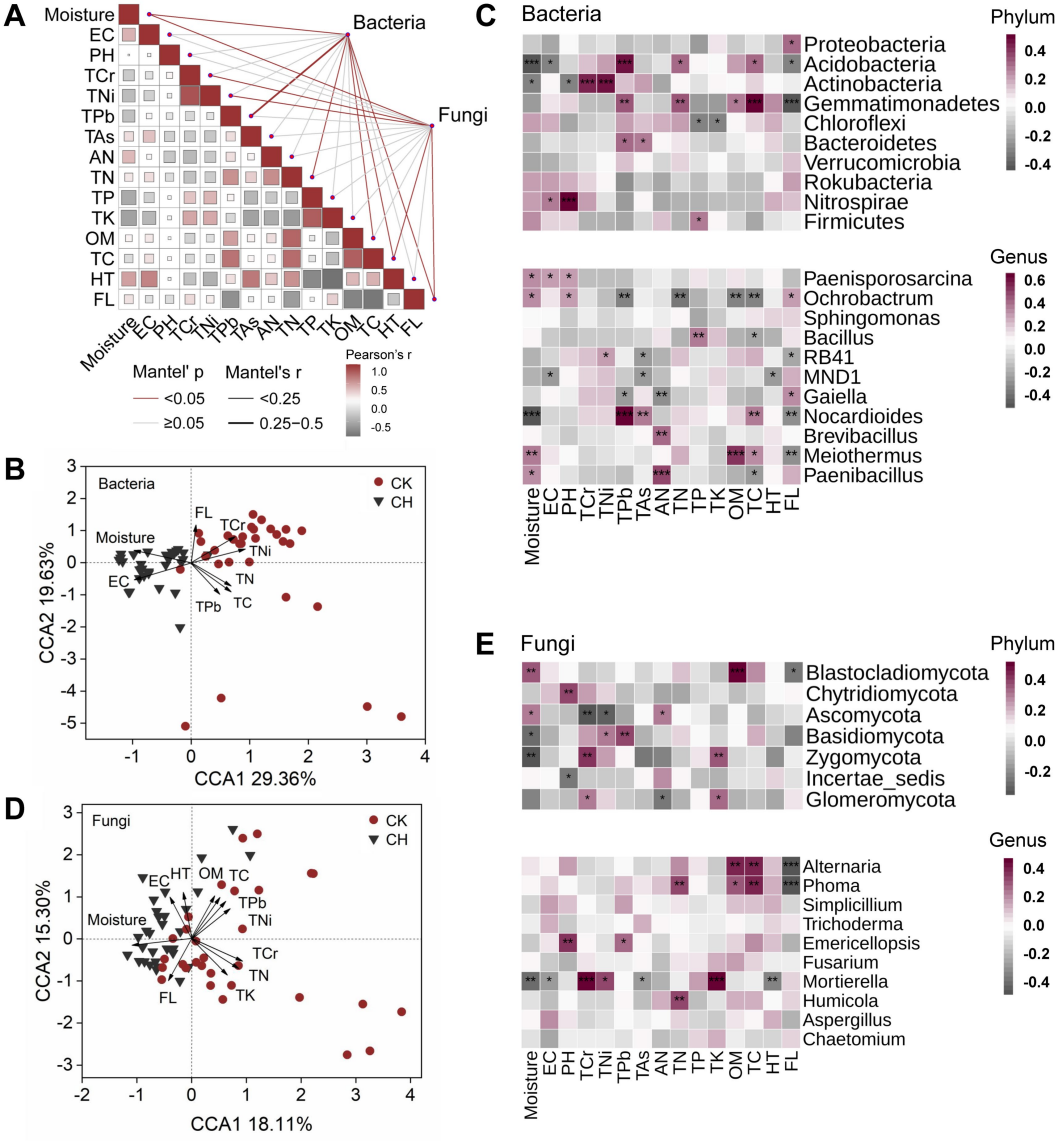
Microbial community structures link to soil physicochemical properties. **(A)** Mantel tests of bacterial and fungal community structures with environmental variables of bare soils (CK) and cement-hardened soils (CH). **(B, D)** CCA of bacterial and fungal communities with environmental variables. **(C, E)** Pearson correlation coefficients of dominant bacterial and fungal phyla and genera with environmental variables indicated by heatmap. EC, electrical conductivity; OM, organic matter; TC, total carbon; TN, total nitrogen; AN, available nitrogen; TP, total phosphorous; TK, total potassium; TCr, total chromium; TNi, total nickel; TPb, total plumbum; TAs, total arsenic; FL, factory location; HT, cement-hardened time. ***p ≤ 0.001, **p ≤ 0.01, and *p ≤ 0.05. CCA, canonical correspondence analysis.

Mantel test revealed that soil moisture, total carbon, and factory location exerted a significant (*p* < 0.05) influence on both bacterial and fungal communities (Figure 6A). Additionally, total Pb, total nitrogen, and organic matter content had strong effects on bacterial community composition, whereas total Cr and total Ni significantly (*p* < 0.05) shaped fungal community structure. Canonical correspondence analysis (CCA) further demonstrated that bacterial and fungal community structures were strongly correlated with soil moisture, electrical conductivity, factory location, and the total levels of Cr, Ni, Pb, nitrogen, and carbon (Figures 6B,D). Furthermore, cement-hardened time, total potassium, and organic matter also significantly (*p* < 0.05) affected the fungal community structure. A heatmap analysis illustrated the influence of soil environmental variables on the relative abundance of dominant bacterial and fungal phyla (Figures 6C,E). Specifically, soil moisture, electrical conductivity, and pH were negatively correlated with the abundances of *Acidobacteriota* and *Actinobacteriota* but positively associated with *Nitrospirae*. Total heavy metal contents in soil positively affected the relative abundances of *Acidobacteria*, *Actinobacteria*, *Gemmatimonadetes*, and *Bacteroidetes*. Furthermore, *Acidobacteria* and *Gemmatimonadetes* exhibited positive correlations with total nitrogen, total carbon, and organic matter. For the fungal phyla, soil moisture and pH significantly affected the relative abundance of dominant fungi. Notably, heavy metal contents contributed more substantial role than soil nutrient components such as available nitrogen and total potassium in driving fungal distribution differences between bare soil and cement-hardened soil. At the genus level, soil moisture, total carbon, and factory location were significantly (*p* < 0.05) correlated with the bacterial abundances of *Ochrobactrum*, *Nocardioides*, and *Meiothermus*. Total plumbum and arsenic contents negatively affected the relative abundances of *Ochrobactrum*, *RB41*, *MND1*, and *Gaiella*. *Meiothermus*, *Bacillus*, *Brevibacillus*, and *Paenibacillus* exhibited positive correlations with organic matter, total phosphorous, and available nitrogen. For the fungal genus, *Mortierella* was positively correlated with soil chromium, total nickel, and total potassium, but negatively correlated with soil moisture and electrical conductivity. Furthermore, soil organic matter and total carbon showed positive correlations with *Alternaria* and *Phoma*.

**Figure 7:**
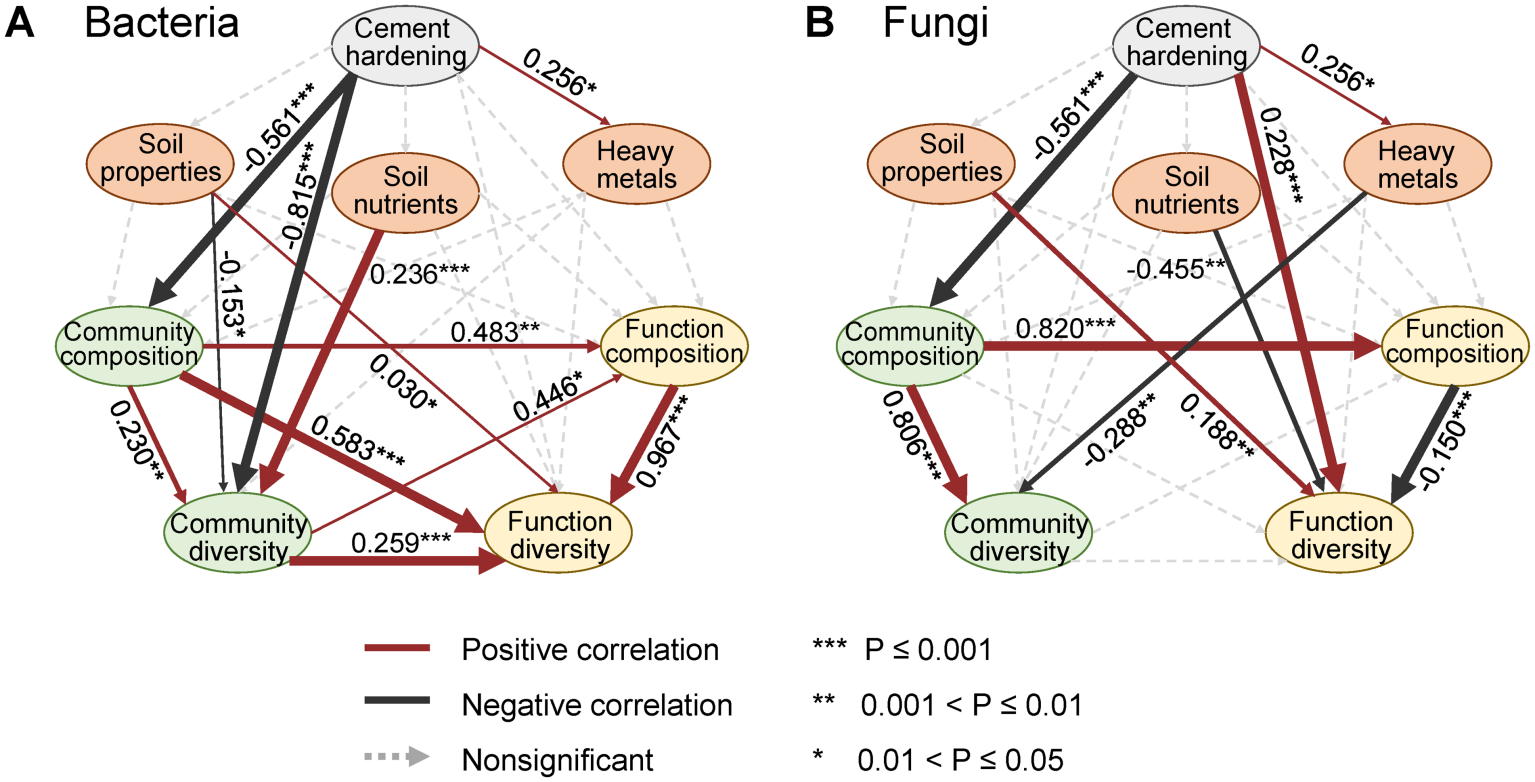
Partial least square path modeling shows the effects of cement hardening on soil environmental variables, microbial community structures, and functions. **(A)** Bacteria. **(B)** Fungi. Red lines and black lines represent the significantly (*p* < 0.05) positive and negative correlations. Dashed lines represent the correlations at a nonsignificant level. ***p ≤ 0.001, **p ≤ 0.01, and *p ≤ 0.05.

Partial least squares path modeling (PLSPM) analysis elucidated the effects of cement hardening on the diversity of bacterial and fungal community structure and function (Figures 7A,B). Cement hardening exerted a direct influence on bacterial community composition and diversity, which in turn indirectly affected bacterial functional composition and diversity. Additionally, soil properties and nutrient levels exhibited significant relationships with both bacterial community diversity and functional diversity. In contrast, cement hardening directly impacted fungal community composition and functional diversity, while indirectly modulated fungal functional composition and community diversity. Furthermore, soil properties and nutrients played a direct role in shaping fungal functional diversity.

## 4 Discussion

Cement hardening significantly altered soil physicochemical properties (Table 1 and Supplementary Figure S1). Increases in soil pH and electrical conductivity (EC) levels were consistent with urbanization-induced changes, likely due to calcium hydroxide release during cement hydration and dissolution of bicarbonate and potassium ions over time (Christiansen et al., 2019). Soil compaction under cement layers reduced pore space and increased bulk density (Cambou et al., 2023). Consequently, the enhanced capillary action facilitated the soil adsorption of dissolved ions from groundwater, leading to elevated soil EC. Contrary to previous studies (Morgenroth et al., 2013; Piotrowska-Długosz and Charzyński, 2015; Hu et al., 2018), sealed soils exhibited higher water content compared to open soils (Table 1), potentially due to sampling during a dry season in abandoned factory sites without irrigation. Heavy metal concentrations were deceased in cement-hardened soils associated with reduced direct anthropogenic inputs, such as soil backfill, construction process, coal combustion, and vehicle exhaust (Li et al., 2001; Ljung et al., 2006; Chen et al., 2010; Hu et al., 2014). However, rainwater erosion likely exacerbated non-point pollution in unsealed soils. Significant reductions in organic matter, total carbon, nitrogen, and potassium in cement-hardened soils were attributed to limited plant litter input and isolation from atmospheric exchange (Pouyat et al., 2006; Churkina et al., 2010). This isolation impeded microbial biomass maintenance by restricting substrate and nutrient inputs (Nannipieri and Eldor, 2009; Wang et al., 2011), while gaseous emissions and aqueous losses further depleted carbon and nitrogen pools (Raciti et al., 2012).

Cement hardening markedly influenced bacterial and fungal community diversity and composition (Figure 2). Fungal alpha-diversity indices (Chao1 and Shannon indices) declined more sharply than bacterial diversity, indicating greater fungal sensitivity to sealing (Figures 2A,B). The greater sensitivity of fungal diversity to cement hardening aligned with broader observations that fungal communities were often more susceptible than bacteria to physical disturbance and heavy metal toxicity in soils. Fungi performed important ecological roles as decomposers, mutualists and pathogens of plants. Nutrient scarcity, elevated pH, limited oxygen, and absence of light under impervious covers likely reduced microbial biomass (Wardle, 1992; Zhao et al., 2012). Beta-diversity significantly differed between cement-hardened (CH) and bare soil (CK) soils, with species replacement accounting for 74.9 and 71.1% of bacterial and fungal compositional dissimilarity, respectively, which demonstrated that the habitats shaped by cement hardening led to the disappearance or replacement of many soil species. Copiotrophic species such as *γ*-*Proteobacteria*, *Bacteroidetes*, and *Actinobacteria* were decreased in CH soils, whereas *Firmicutes* was favored under impervious conditions, which showed its resistant tolerant to environmental extremes (Mendes et al., 2015).

The increased bacterial and fungal network complexity under cement hardening contrasted with the reports of simplified microbial networks in sealed urban turfgrass and compacted forest soils (Zhang et al., 2020; Liu et al., 2023). This discrepancy might stem from the unique baseline conditions of the industrial sites. Unlike those ecosystems, the bare industrial soils experienced high spatial heterogeneity and chronic metal stress, leading to fragmented niches and unstable networks. The topology of co-occurrence networks expressed the direct and indirect relationships between microbes and environments (Guseva et al., 2022). Higher link number, connectivity, average degree, clustering coefficient, and centralization, alongside lower modularity in CH soils suggested the enhanced microbial interactions under sealing. This may reflect weak spatial connectivity and niche fragmentation in bare industrial soils (Bissett et al., 2013; Xue et al., 2020), where variable physicochemical conditions (Supplementary Figure S1) and anthropogenic disturbances led to disjointed microbial niches (Delbecque et al., 2022). Dominant copiotrophic taxa of *Firmicutes* and *Ascomycota* in CH soils adapted to resource-rich conditions potentially facilitated the direct and indirect interactions with oligotrophic species (Morris et al., 2013), increasing the network complexity (Thompson and Kao-Kniffin, 2019). Bacterial network robustness was lower in CK soils, supporting the notion that fragmented networks are less stable (Mougi and Kondoh, 2012). Conversely, fungal networks in CH soils exhibited decreased robustness under targeted removal, likely due to strong environmental filtering and heterogeneous selection (Figure 4), which increased interaction complexity but compromised stability (Karimi et al., 2017).

Cement hardening reduced the diversity of microbial ecological functions by limiting inputs of plant and animal residues and accelerating nutrient depletion (Figures 5A,B) (Degens et al., 2000). Soil compaction further diminished porosity, aeration, and water availability, impairing metabolic potential (Figures 5C,D) (Pengthamkeerati et al., 2011). For instance, anaerobic processes such as fermentation, nitrate and sulfate reduction, and methanogenesis were enriched in CH soils (Figure 5E), while saprotrophic capabilities (e.g., wood, litter, and dung decomposition) declined (Figure 5F). The shift towards anaerobic metabolism was a common functional signature in environments with restricted gas exchange, analogous to trends observed in waterlogged paddy soils. Conversely, the decline in saprotrophic potential reflected a broader pattern of functional erosion in urban soils isolated from plant-derived organic matter inputs. From the perspective of microbial survival, under habitat fragmentation and anthropogenic pressure, essential metabolic pathways, including transcription, nucleotide metabolism, and amino acid metabolism were reinforced in CH soils (Peng et al., 2020), potentially supporting microbial adaptation and growth (Yi et al., 2022). Notably, the enrichment of potential pathogens and reduction in xenobiotics degradation capacity in CH soils highlight health risks associated with soil resealing in abandoned industrial areas.

Interactions and variations in environmental variables played key roles in shaping microbial community structure in diverse ecosystems (Drenovsky et al., 2004; Berg and Smalla, 2009). Mantel tests and canonical correlation analysis (CCA) revealed that moisture, nutrients, and heavy metals were related to bacterial and fungal communities across soil cover types (Figures 6A–D). Water stress could alter substrate availability and modify microbial composition (Goldfarb et al., 2011; Hueso et al., 2012), while nutrient levels and heavy metal contamination critically influenced microbial biomass, activity, and functional diversity in urban settings (Zhao et al., 2013; Chen et al., 2014; Xie et al., 2016). The observed reduction in total heavy metal contents under cement hardening likely exerted a profound selective pressure on the microbial community. Heavy metals could directly inhibit sensitive microbial taxa through oxidative stress and enzyme disruption. Our extended analysis revealed that heavy metal concentrations explained a significant portion of the microbial community variation (Figure 6), independent of nutrient levels. This suggested that the alleviation of metal stress in CH soils, possibly due to isolation from fresh anthropogenic inputs, might have facilitated the proliferation of metal-tolerant groups (e.g., some *Firmicutes* lineages known for metal resistance) and contributed to the shift in community assembly towards deterministic selection (Figure 4). The differential sensitivity of fungi, which often showed higher susceptibility to heavy metals than bacteria, could be an additional factor underlying their sharper diversity loss in CH soils. However, no single variable predominated in predicting community structure (Xu et al., 2014), given the multifactorial influence of anthropogenic activities, soil temperature, texture, and hydraulic properties (Chen et al., 2005; Karim et al., 2014). Causal relationships among these factors warrant further experimental validation.

Our experimental framework explicitly incorporated spatial heterogeneity as a constitutive element of the urban soil environment, rather than treating it as mere statistical noise. Crucially, we observed consistent directional shifts in microbial communities across all sites, despite pronounced disparities in baseline industrial pollution. These demonstrated that cement hardening acted as a powerful, overarching ecological filter capable of overriding certain site-specific initial conditions. Simultaneously, the observed heterogeneity provided preliminary evidence for context-dependent modulation of the sealing effect. For instance, more severe nutrient depletion at particular sites appeared to intensify the loss of copiotrophic bacterial taxa. Therefore, this study served a universal signal of cement-hardening impact on soil microbiomes and established a targeted foundation for future mechanistic investigations.

Based on the above discussion, this study offers significant practical applications for urban planning and environmental management. Soil cement hardening in abandoned industrial sites reduces direct heavy metal contamination, but simultaneously degrades soil ecological functions and increases pathogenic risks. Consequently, urban renewal projects should thoroughly evaluate the long-term ecological impacts of impervious surfaces. A key recommendation is advocating for permeable pavements in suitable locations to maintain essential air-water exchange, thereby preserving microbial activity and nutrient cycling. Furthermore, specific microbial indicators such as *Firmicutes* dominance and reduced saprotrophic potential can serve as bio-monitoring tools to assess the sealed soil health and guide targeted remediation efforts in contaminated areas. Future research should focus on advancing mechanistic understanding and developing intervention strategies. First, long-term in-situ experiments are necessary to clarify the causal relationships between environmental variables and microbial community stability. Second, expanding research to diverse urban settings and climatic conditions will help generalize these findings. Finally, integrating molecular data with physical-hydrological modeling will be crucial for predicting microbial responses to different urban soil management practices. Although our multi-site, paired design was robust for detecting the general ecological impact of cement hardening, it inherently averaged over fine-scale, site-specific management practices (e.g., crack presence, cleaning routines). Future studies focusing on single sites could powerfully investigate how such micro-variations modulate the sealing effect we describe here.

## 5 Conclusion

This study elucidated the profound impact of cement hardening on the soil microbiome within industrial areas, moving beyond descriptive taxonomy to provide an ecosystem-level perspective. Cement hardening reduced exogenous nutrient inputs and heavy metal accumulation, thereby markedly altering the diversity and composition of soil bacterial and fungal communities. This cement sealing increased the size and complexity of microbial co-occurrence networks but decreased the stability of fungal network. Concurrently, microbial community assembly shifted towards deterministic processes, with heterogeneous selection becoming the dominant driver of phylogenetic variation. These structural shifts corresponded to a change in functional potential, characterized by an enrichment of respiratory bacteria but a decline in the inferred capacities for xenobiotic degradation and fungal saprotrophic functions. Collectively, our results provided novel mechanistic insights into anthropogenic soil sealing. They offered concrete, evidence-based guidance for sustainable industrial land management: minimizing unnecessary soil sealing, designing spatial mosaics of sealed and unsealed areas, and strategically preserving functional soil microbiomes should be key considerations in the ecological planning of industrial parks.

## Data Availability

The original contributions presented in the study are included in the article/Supplementary material, further inquiries can be directed to the corresponding authors.
