# Supplementary material for: Cement hardening reshapes soil microbial diversity, network stability and ecological functions of industrial area
Source: Front Microbiol. 2026 Feb 12;17:1750259. doi: 10.3389/fmicb.2026.1750259 (PMC12936048; doi:10.3389/fmicb.2026.1750259)
Supplement: Supplementary file 1 [file Data_Sheet_1.docx]

***Supplementary Material***

**Cement hardening reshapes soil microbial diversity, network stability and ecological functions of industrial area**

Xiaodong Hao^1^, Hui Li^1^, Xiaomeng Wang^1^, Ping Zhu^1^, Aide Sun^1^,

Qishi Luo^1*^, Xu Zhang^2^, Zhiqun Chen^2*^, Xueduan Liu^3^

^1^ College of Resources and Environment, Linyi University, Linyi 276000, China

^2^ College of Life Science, Linyi University, Linyi 276000, China

^3^ School of Minerals Processing and Bioengineering, Central South University, Changsha 410083, China

***Correspondence:**

qsluo99@126.com (Q. Luo)

[chenzhiqun@lyu.edu.cn](mailto:chenzhiqun@lyu.edu.cn) (Z. Chen)

**FIGURE S1.** Principal component analysis (PCA) shows the difference of physicochemical properties between bare soils (CK) and cement-hardened soils (CH) based on all measured and homogenized soil variables. Similarity test (Anosim) showing the significant (P ≤ 0.05) difference.


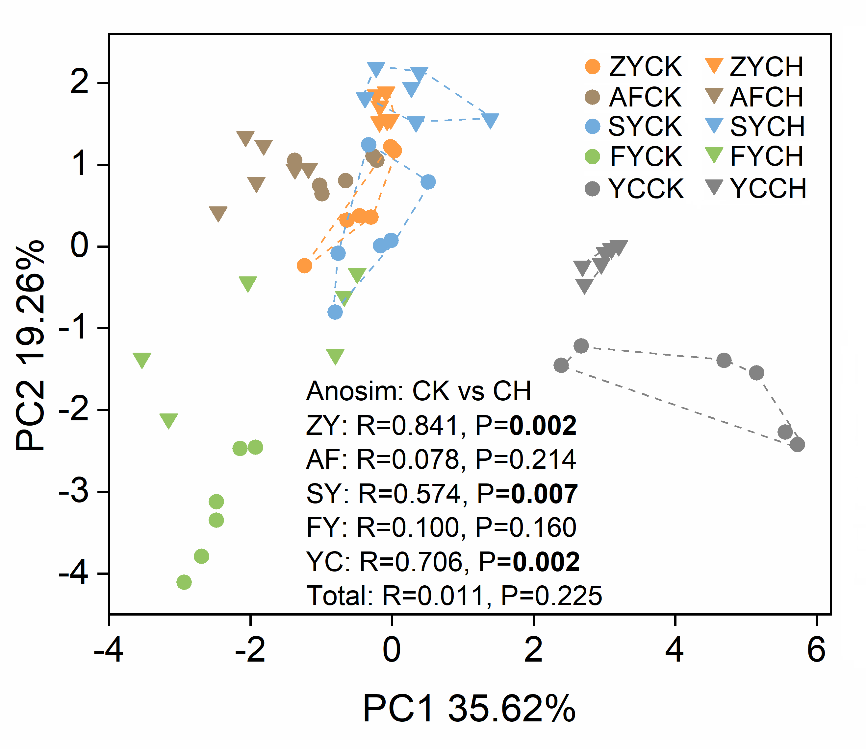


**TABLE S1.** Soil physicochemical properties of bare soils (CK) and cement-hardened soils (CH) in five factory sites (n = 6).

| Item | ZYCK | ZYCH | AFCK | AFCH | SYCK | SYCH | FYCK | FYCH | YCCK | YCCH |
| --- | --- | --- | --- | --- | --- | --- | --- | --- | --- | --- |
| Moisture, % | 15.5±1.5 | **17.6±1.6** | 13.1±2.4 | **19.9±2.2** | 15.2±3.0 | **18.1±1.4** | 7.7±2.6 | **16.7±6.5** | 5.8±1.5 | **11.2±0.7** |
| EC, μS/cm | **280.0±28.0** | 187.8±29.4 | 513.7±255.7 | 669.5±366.0 | 547.0±96.3 | **813.0±106.5** | 455.3±189.8 | 498.2±100.9 | 188.0±15.4 | **290.8±39.9** |
| pH | **7.9±0.1** | 7.8±0.1 | 7.7±0.1 | 7.7±0.2 | 7.8±0.2 | **8.4±0.5** | 7.9±0.1 | **8.2±0.2** | 7.8±0.1 | 8.1±0.5 |
| OM, g/kg | **18.0±5.2** | 9.3±1.2 | 15.5±3.6 | **20.8±1.5** | **23.0±7.3** | 8.5±2.2 | 42.5±10.6 | 50.9±27.6 | **13.6±9.5** | 4.0±1.0 |
| TC, g/kg | **14.9±4.5** | 5.7±0.7 | 11.5±3.7 | 12.6±2.1 | **29.6±9.1** | 6.5±2.6 | 60.0±21.4 | 47.3±11.6 | **8.6±2.8** | 2.3±0.6 |
| TN, g/kg | **1.1±0.3** | 0.6±0.1 | 0.9±0.1 | **1.3±0.1** | **0.8±0.3** | 0.5±0.1 | 1.5±0.2 | 1.3±0.3 | **0.4±0.1** | 0.3±0.1 |
| AN, mg/kg | 79.3±10.4 | 82.5±10.8 | 54.3±17.6 | **103.3±31.7** | 54.5±10.1 | 58.3±12.9 | 80.0±8.6 | 65.7±18.4 | 42.3±9.3 | 52.3±15.8 |
| TP, g/kg | **0.5±0.1** | 0.3±0.1 | 0.5±0.1 | **0.5±0.1** | 0.5±0.1 | 0.5±0.1 | 0.6±0.1 | 0.6±0.2 | 1.0±0.1 | 1.2±0.2 |
| TK, g/kg | **18.6±0.4** | 17.4±0.3 | **17.4±0.5** | 15.9±0.6 | 19.1±1.2 | 18.3±0.8 | 17.0±1.0 | 16.9±0.8 | 32.7±4.4 | 30.4±1.8 |
| TCr, mg/kg | 79.4±4.0 | 75.3±6.7 | 79.1±8.2 | **96.8±3.6** | 179.7±95.8 | 155.5±76.9 | 64.9±8.3 | 65.7±7.8 | 352.9±232.0 | 99.4±30.2 |
| TNi, mg/kg | 30.4±1.9 | **35.3±3.6** | 46.2±5.8 | 50.6±0.8 | 60.6±13.0 | 48.9±15.4 | 31.2±5.3 | 26.0±2.5 | **768.6±638.6** | 50.8±15.3 |
| TPb, mg/kg | 28.1±1.7 | 26.1±1.7 | 30.4±2.1 | 31.7±1.2 | **28.4±2.1** | 25.2±1.4 | **53.3±7.5** | 31.6±6.0 | 28.5±1.6 | 29.4±0.7 |
| TAs, mg/kg | 6.8±0.5 | **8.1±0.6** | **19.1±4.5** | 11.4±1.4 | 10.4±0.7 | 9.8±1.0 | 10.6±4.1 | 8.4±3.2 | 4.1±1.2 | 4.1±1.0 |

EC, electrical conductivity; OM, organic matter; TC, total carbon; TN, total nitrogen; AN, available nitrogen; TP, total phosphorous; TK, total potassium; TCr, total chromium; TNi, total nickel; TPb, total plumbum; TAs, total arsenic. Bold fonts in CK and CH columns represent the significantly (P ≤ 0.05) higher means according to Wilcoxon signed-rank test.
